# Comparing the locking screw direction of three locking plates for lateral clavicle fractures: a simulation study

**DOI:** 10.1186/s12891-021-04697-5

**Published:** 2021-09-21

**Authors:** Shingo Abe, Kota Koizumi, Tsuyoshi Murase, Kohji Kuriyama

**Affiliations:** 1 Toyonaka Municipal Hospital, 4-14-1 Shibahara, Toyonaka, Osaka, 560-8565 Japan; 2 Department of Orthopaedic Surgery, Osaka University Graduate School of Medicine, 2-2 Yamadaoka, Suita, Osaka, 565-0871 Japan

**Keywords:** Locking plate, Lateral clavicle fractures, Screw direction, Lateral fragment size

## Abstract

**Background:**

The locking plate is a useful treatment for lateral clavicle fractures, however, there are limits to the fragment size that can be fixed. The current study aimed to measure the screw angles of three locking plates for lateral clavicle fractures. In addition, to assess the number of screws that can be inserted in different fragment sizes, to elucidate the size limits for locking plate fixation.

**Methods:**

The following three locking plates were analyzed: the distal clavicle plate [Acumed, LLC, Oregon, the USA], the LCP clavicle plate lateral extension [Depuy Synthes, LLC, PA, the USA], and the HAI clavicle plate [HOMS Engineering, Inc., Nagano, Japan]. We measured the angles between the most medial and lateral locking screws in the coronal plane and between the most anterior and posterior locking screws in the sagittal plane. A computer simulation was used to position the plates as laterally as possible in ten normal three-dimensional clavicle models. Lateral fragment sizes of 10, 15, 20, 25, and 30 mm were simulated in the acromioclavicular joint, and the number of screws that could be inserted in the lateral fragment was assessed. Subsequently, the area covered by the locking screws on the inferior surface of the clavicle was measured.

**Results:**

The distal clavicle plate had relatively large screw angles (20° in the coronal plane and 32° in the sagittal plane). The LCP clavicle lateral extension had a large angle (38°) in the sagittal plane. However, the maximum angle of the HAI clavicle plate was 13° in either plane. The distal clavicle plate allowed most screws to be inserted in each size of bone fragment. For all locking plates, all screws could be inserted in 25 mm fragments. The screws of distal clavicle plate covered the largest area on the inferior surface of the clavicle.

**Conclusions:**

Screw angles and the numbers of screws that could be inserted in the lateral fragment differed among products. Other augmented fixation procedures should be considered for fractures with fragment sizes < 25 mm that cannot be fixed with a sufficient number of screws.

**Supplementary Information:**

The online version contains supplementary material available at 10.1186/s12891-021-04697-5.

## Background

Displaced lateral clavicle fractures have a high risk of delayed or non-union [1]. Conservative therapy results in non-union in approximately 11.5% of patients with lateral clavicle fractures [2]. Therefore, surgical treatment for displaced lateral clavicle fractures is preferred [3]. There are numerous surgical options for fixing lateral clavicle fractures. The lateral clavicle locking plate osteosynthesis that does not overlap the acromioclavicular joint (ACJ), hook plate osteosynthesis that spans the ACJ, and reconstruction of the coracoclavicular ligament [4–6]. Locking plate fixation is usually used in patients with a relatively large lateral fragment because a small fragment cannot take enough screws to achieve robust fixation. However, the minimum fragment size that can be fixed with a locking plate alone has not been fully elucidated. Lateral clavicle locking plates each have a unique fixed screw angle that may affect the size of fixable bone fragments. The screw angles of the locking plates vary according to the products; therefore, knowledge of their screw angles is useful for surgical planning. We hypothesized that the angles between the screw and plate and the number of screws that could be inserted into the fragments would vary among the available products. This study aimed to measure the three-dimensional (3D) screw angles of three locking plates for lateral clavicle fractures and to quantify the numbers of screws that could be inserted for each fragment size in lateral clavicle fractures.

## Methods

### Locking plate model and simulation

**Fig. 1:**
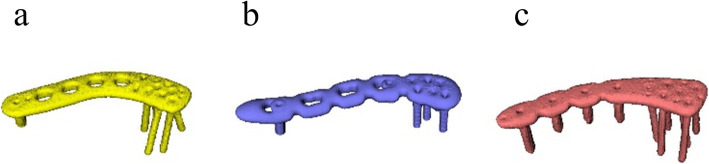
Three locking plate models were shown. (a) Distal clavicle plate [Acumed, LLC, Oregon, the USA]; (b) LCP clavicle plate lateral extension [Depuy Synthes, LLC, MA, the USA]; and (c) HAI clavicle plate [HOMS Engineering, Inc., Nagano, Japan]

We conducted a simulation study using 3D plate models and in vivo 3D clavicle bone models. Three locking plates for lateral clavicle fractures (distal clavicle plate [Acumed, LLC, Oregon, the USA], LCP clavicle plate lateral extension [Depuy Synthes, LLC, PA, the USA], and HAI clavicle plate [HOMS Engineering, Inc., Nagano, Japan]) were assessed. We selected these clinically available plates and we were familiar with their use. Moreover, the distal clavicle plate and LCP clavicle plate are widely used worldwide [7]. The number of locking screws on the lateral end and their orientation varied according to the products. The distal clavicle plate has a relatively low profile and eight radially arranged locking screws on the lateral end. The LCP clavicle plate lateral extension has six locking screws on the lateral end, with three screws angled forward and three backward. The HAI clavicle plate has six locking screws angled relatively straight (Fig. 1).

To perform simulation, we created 3D model of the plate using a computed tomography (CT) and computer software (Bone Viewer; Orthree Co., Ltd., Osaka, Japan) [8]. The 3D model accuracy of this system was reported to be 0.46 mm [9]. Ten patients (5 men and 5 women) with proximal humeral fractures admitted at the authors’ institution between July 2018 and July 2019 underwent preoperative CT scans from the entire clavicle to the humerus for pre-surgical planning. All patients provided informed consent. The authors created 3D models of a normal clavicle bone the same way the plate models were created [8]. When the clavicle was on the right, it was transposed to the left using different software (Bone Simulator; Orthree Co., Ltd., Osaka, Japan). The average clavicular length was 148 mm (range, 132–167 mm), while the average midshaft diameter was 11.5 mm (range, 8.9–13.6 mm). The authors performed a computer simulation using the Bone Simulator to locate the locking plates as far laterally on the clavicles as possible to avoid encroaching on the ACJ. The authors also ensured that the medial shaft of the plate did not deviate from the clavicle. Since, during surgery, the plate is usually bent to fit the curvature of the individual clavicle, fitting the plate to the clavicle during simulation prioritized the lateral end, not the clavicle midshaft. The simulations were performed to minimize the gap between the lateral end of the plates and the clavicle. Two orthopedic surgeons, each with over 10 years of experience, independently performed the simulation twice at 4-week intervals. Therefore, 40 simulations were performed for each locking plate. To precisely identify the plate’s position, the distance between the ACJ and lateral end of the plate was measured.

### Screw angles of the locking plates for lateral clavicle fractures

**Fig. 2:**
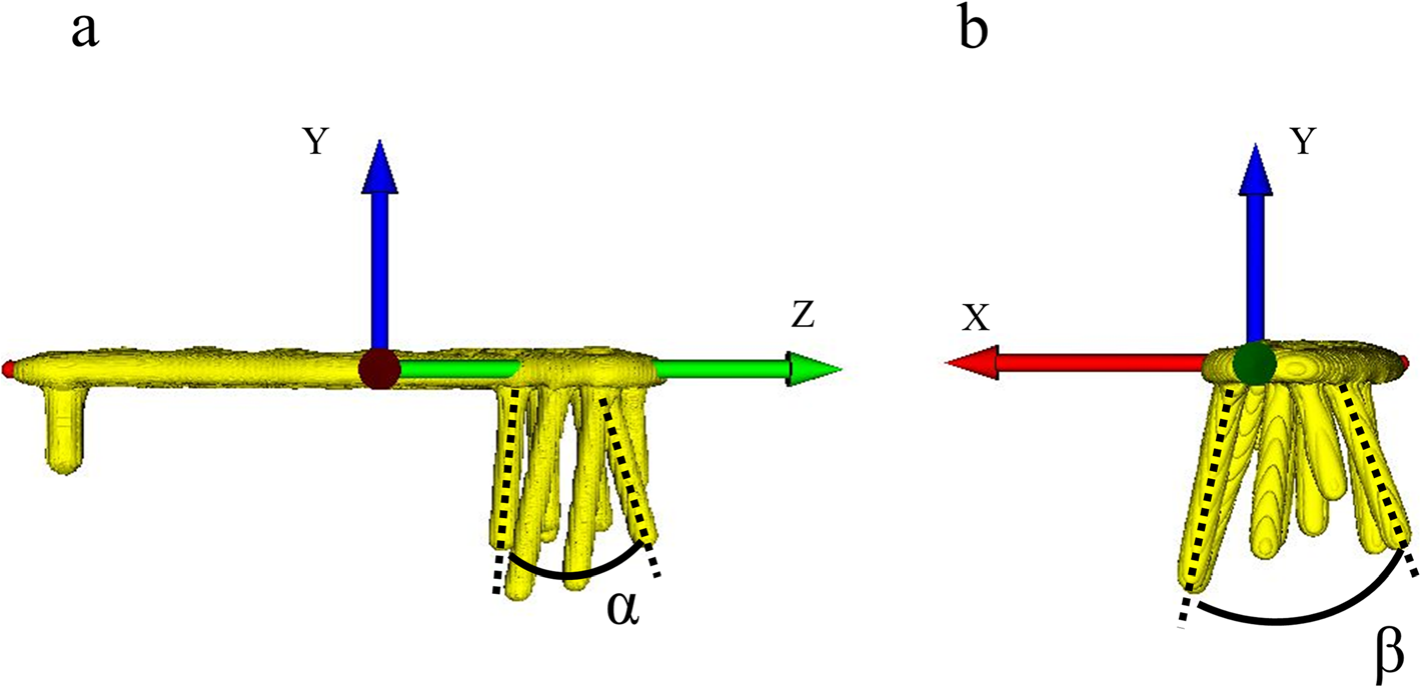
Screw angle between the most lateral and medial screws, defined as α angle, in the YZ plane (coronal plane), and the screw angle between the most anterior and posterior screws, defined as β angle, in the XY plane (sagittal plane)

The orthogonal coordinate system was applied to assess the 3D screw angles of the plates. Details of the coordinate system are given in Supplementary Material 1. The screw angle between the most lateral and medial screws, defined as the α angle, in the YZ plane (coronal plane) and the screw angle between the most anterior and posterior screws, defined as the β angle, in the XY plane (sagittal plane) were measured (Fig. 2).

### Number of screws inserted in the lateral clavicle fragment and the area covered with locking screws on the inferior surface of the fragment

To ensure repeatable measurements, the authors also applied the orthogonal coordinate system for each clavicle based on the International Society of Biomechanics recommendations [10]. Details of the coordinate system for the clavicle are given in Supplementary Material 1. The distal end of the clavicle was marked at 5 mm intervals along the Z-axis from the ACJ. Then, a lateral fragment size of 10, 15, 20, 25, and 30 mm was simulated in the ACJ. Because the coracoclavicular ligament attached to the clavicle’s inferior surface was between 8 and 9 mm and 44–47 mm from the ACJ (trapezoid ligament 8–26 mm; conoid ligament 26–47 mm), Craig type 1, 2B, 3, and 5 fracture lines could be simulated [11–14]. Craig type 1 and 3 fractures had a fracture line within 10 mm of the ACJ. Craig type 2B and type 5 fractures involved ligament injury. The authors counted the number of screws completely inserted in each size of bony fragment. To quantify the area covered by all screws on the inferior surface of the clavicle, a line connecting the adjacent screws on the inferior surface of the clavicle was drawn, and the enclosed area was measured.

### Data and statistical analyses

Each examiner performed the plate-setting simulation twice. To assess the simulation’s repeatability, the authors conducted two one-sided tests for the distance between the ACJ and lateral end of the plates. The mean absolute difference was calculated with a 95% confidence interval between simulations 1 and 2 for each examiner and between them. The numbers of screws were compared using the Steel–Dwass test, and the average area covered by the locking screws was compared using Tukey’s Honestly Significant Difference test (*p* <  0.05). Statistical tests were performed using JMP Pro 14.0 (SAS Institute Inc., Cary, NC).

## Results

The distal clavicle plate had eight locking screws and relatively large α and β angles (20° and 32°, respectively). The LCP clavicle lateral extension had six locking screws with a large β angle (38°) and small α angle (12°). The HAI clavicle plate had six locking screws with small α (3°) and β (12°) angles.

The distribution of intraobserver and interobserver plate position simulations is shown in Table 1. The plate position simulation was performed with a variation within 1.5 mm and two one-sided tests indicated that most simulations were performed within 3 mm in the equivalence test. Owing to high repeatability, the authors described the average of all 40 simulations in the results.

**Table 1:** Mean absolute difference with 95% confidence interval (CI) between the simulation trials and *p* value of two one-sided test (TOST) with a threshold of 3 mm

	Distal clavicle plate		LCP clavicle plate lateral extension		HAI clavicle plate	
	Mean difference (mm) [95% CI]	*p* value	Mean difference (mm) [95% CI]	*p* value	Mean difference (mm) [95% CI]	*p* value
Between simulations 1 and 2 for examiner 1	0.56 [−0.04–1.16]	0.031	1.76 [−0.35–3.87]	0.156	0.40 [−0.15–0.95]	0.007
Between simulations 1 and 2 for examiner 2	0.76 [0.18–1.33]	0.029	0.86 [0.19–1.52]	0.055	0.18 [−0.19–1.35]	0.003
Between examiners 1 and 2	0.54 [0.05–1.02]	0.002	0.88 [0.23–1.53]	0.012	0.18 [−0.15–0.94]	< 0.001

*CI* confidence interval

**Fig. 3:**
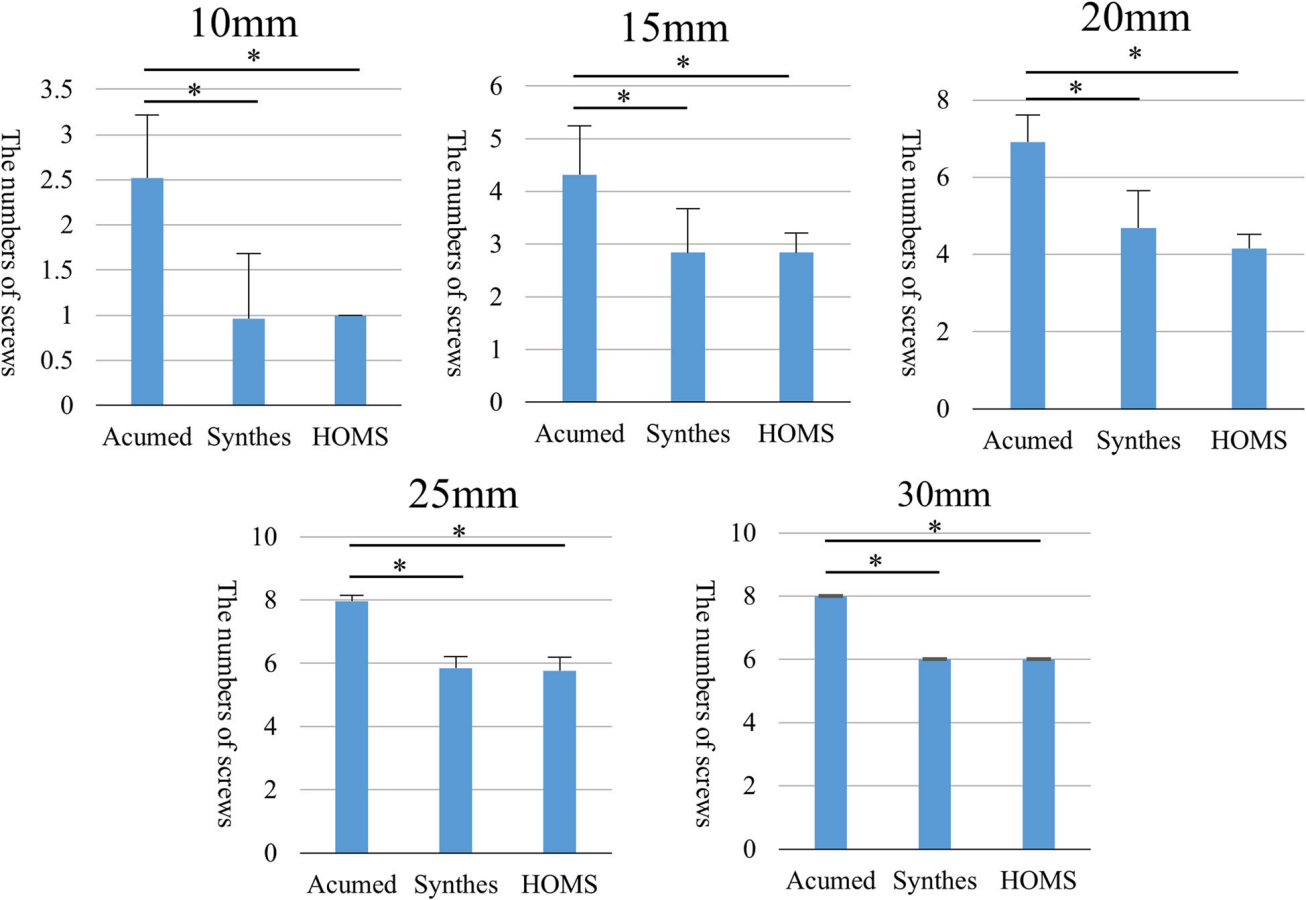
Average number of screws that could be completely inserted in the lateral fragment with standard deviation (error bar). Lateral fragment was simulated 10, 15, 20, 25, and 30 mm from the ACJ. An average of 40 simulations was performed. Asterisk indicates statistically significant difference, *p* <  0.05

**Fig. 4:**
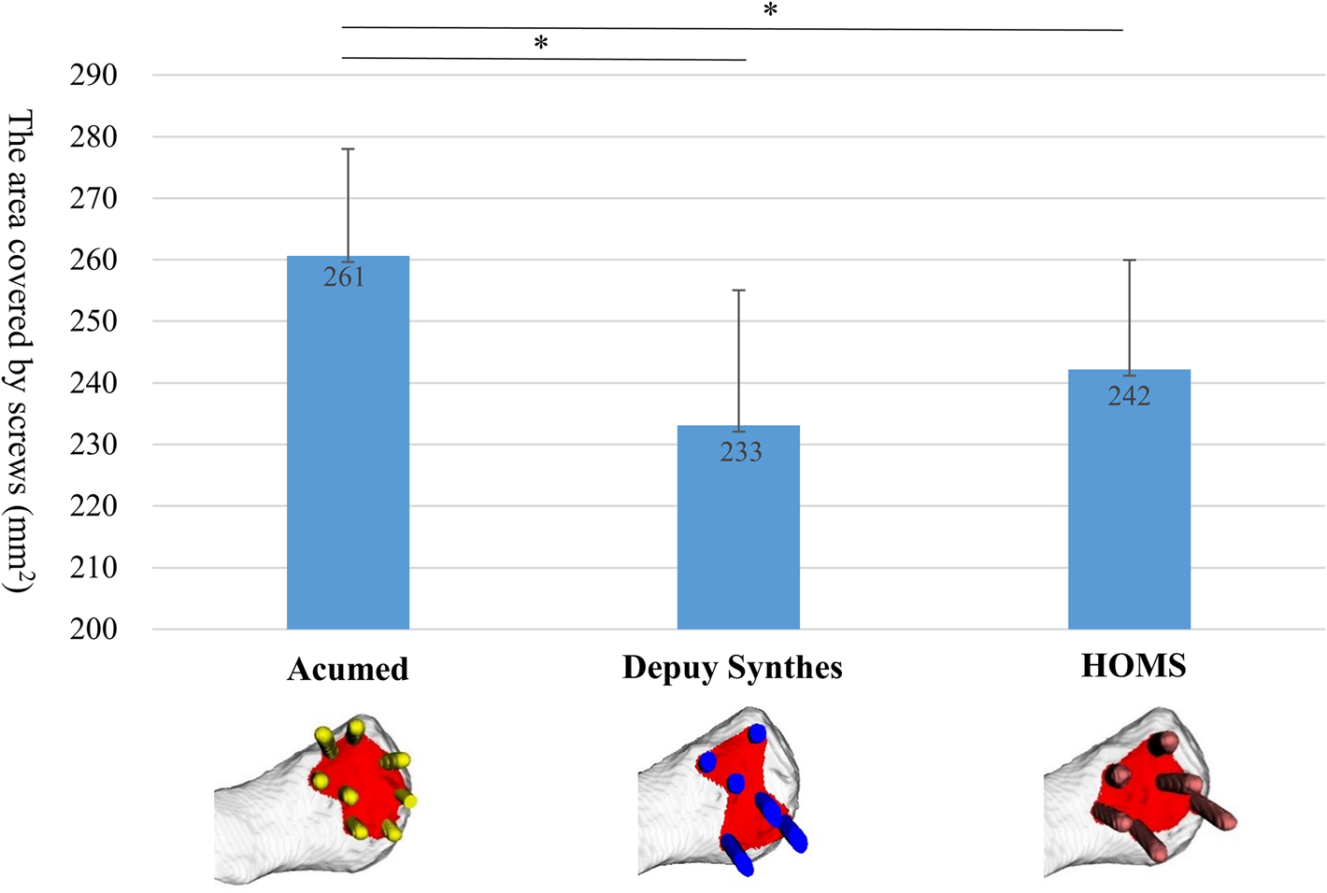
Average covered area by locking screws on the inferior surface of the clavicle with standard deviation (error bar). An average of 40 simulations was performed. Asterisk indicated statistically significant difference, *p* < 0.05

The average number of screws for each lateral fragment size is shown in Fig. 3. The distal clavicle plate could be inserted with many screws in each fragment size. For all locking plates, all screws could be inserted in the 25 mm fragments. The screw-covered area is shown in Fig. 4. The distal clavicle plate could cover the largest area of all plates on the inferior surface of the clavicle within 25 mm of the ACJ.

## Discussion

The authors assessed the 3D screw angles, the number of screws that could be completely inserted in each size of bony fragment, and the screw-covered area on the inferior surface of the lateral clavicle fractures for three locking plates. The distal clavicle plate could fix a relatively small fragment because it had the largest number of screws that could be inserted in each fragment size. It had large α and β angles, and the eight locking screws at the lateral end could capture a wide area on the clavicle’s inferior surface. The LCP clavicle lateral extension had large β angles, indicating the screws were widely spread in the anteroposterior direction. The HAI clavicle locking plate comprised six locking screws with a relatively straight alignment which covered an area as large as the LCP clavicle lateral extension plate.

The displaced lateral clavicle fracture has a high risk of non-union if managed conservatively [1, 2]. Although several surgical methods are available, including K-wire fixation, hook plate fixation, coracoclavicular ligament reconstruction, and lateral clavicle locking plate fixation, there is no consensus on the most suitable method [3]. Hook plate fixation has a good union rate. However, complications, such as acromial osteolysis, subacromial impingement, and ACJ arthrosis, may occur [15, 16]. Coracoclavicular ligament reconstruction has achieved robust fixation in a biomechanical study [17]. This procedure would be useful for fractures with coracoclavicular ligament injury and a comminuted inferior surface, where the screws could not fix the fragments [18]. However, it has been reported that ligament reconstruction increases the risk of non-union for fragment sizes over 3 cm [19]. The lateral clavicle locking plate has a higher union rate and lower complication rate than the hook plate; however, it cannot achieve strong fixation with a small lateral fragment using the locking plate because of the limited number of screw insertions. According to an anatomical cadaver study, the coracoclavicular ligaments (trapezoid ligament and conoid ligament), which contribute to the stability of the lateral end of the clavicle, begin 8–9 mm from the ACJ and end 44–47 mm from the ACJ (trapezoid ligament, 8–26 mm; conoid ligament, 26–47 mm) [11, 12]. The coracoclavicular ligament is important and contributes a greater constraint with larger displacements [20]. Therefore, the displaced lateral clavicle fracture with a lateral fragment < 9 mm, such as Craig types 1 and 3, may be difficult to fix with a locking plate alone due to the absence of the coracoclavicular ligament on the lateral fragment [13, 14]. Because 2 or 3 screws at most could be inserted in lateral fragments < 10 mm in this study, hook plate fixation would be the best option for fractures of this size. Displaced fractures with lateral fragments > 10 mm, such as Craig type 2B and 5 involving the coracoclavicular ligament, need stabilization between the fragment with the ligament and the lateral fragment. Although there is no biomechanical data regarding sufficient locking plate screw numbers for lateral clavicle fractures, more than three-quarters of screw holes should be occupied by locking screws on distal fragments when using locking compression plates for limb fractures [21]. For lateral clavicle fragments that are > 20 mm in size, the distal clavicle plate, and LCP clavicle lateral extension could insert more than three-quarters of their maximum number of screws into the fragments. If the lateral fragment is smaller than 20 mm, an additional procedure should be considered, such as cerclage wiring between the clavicle and plate, suture augmentation between the coracoid and plate, or anchor placement into the coracoid passing and tying to the plate [22–25]. Regarding screw angles, biomechanical studies have reported on the relationship between screw angles and fixation strength; however, there is no strong consensus. Wähnert et al. reported that pull-out forces and axial stiffness were higher in screws oriented within 10° divergence than 20° [26]. Robert et al. reported significantly higher fixation strength in divergent screw angles of 20° and 30° compared to a 0° angle [27]. Our results showed that locking plates with large screw angles could insert more screws in the lateral fragment and cover a larger inferior surface area. Regarding the relationship between screw angles and fixation strength, it is uncertain that the area covered by the screws relates to fixation strength; however, the larger the area covered by the screws, the more the insertion area of the coracoclavicular ligament would be covered.

The present study had some limitations. First, this is a simulation study. It was easier to set the plate more laterally in the simulation than in a clinical situation because there were no obstacles. Moreover, biomechanical data, such as pull-out force or fixation strength between the bone and locking plate fixation, were not considered. Soft tissue injury and osteoporosis that could affect the stability of the fixation were not represented in this simulation model. Second, the angle of fixation of the lateral locking screws in some plates varied. The surgeons could choose the screw angle based on the intraoperative situation when using different locking plates; however, the differences in the locking plates were not assessed in this study. If we had been able to make a 3D model of a variable locking plate and indicate the screw insertion area using the software, we would have provided a more comprehensive strategy for fracture fixation. Third, clavicles have anatomical variations. Although the authors could not cover all clavicle size variations, they investigated ten male and female clavicles, and the authors believe they can generalize their results. Despite these limitations, the authors could elucidate the relationship between fragment size and locking plate fixation. We could also perform pre-surgical simulations with these plate models and individual fracture models using the software to explore the best-fit locking plate. Further biomechanical studies or finite element analysis would clarify the strength of locking plate fixation. The authors believe that this study can help surgeons select implants and plan their surgical strategy for lateral clavicle fractures.

## Conclusions

The screw angles and the numbers of screws that could be inserted in the lateral fragment differed among products. Although all locking plates could insert all their locking screws within 25 mm fragments, the distal clavicle plate could insert the largest number of screws and cover the largest area in the lateral fragment of the plates. The surgeon should be familiar with the characteristics of the plates. Other augmented fixation procedures should be considered for fractures with fragment size < 25 mm that cannot accept enough screws.

## Supplementary Information

**Additional file 1: Supplementary material 1**. The coordinate system of the plate. Three points on the plates were assessed: the medial and lateral end points of the plate with its midpoint of thickness, the midpoint of the length with dorsal rim, and midpoint of the thickness of the plate. The basal plane that passes through these three points was created. The Z axis passed through the medial and lateral points and pointed toward the lateral side of the plate on the basal plane. The X axis pointed toward the volar side of the plate on the basal plane perpendicular to the Z axis. The Y axis was perpendicular to both the X and Y axes and pointed toward the cranial side. The coordinate system of the clavicle. The Z axis connected the most anterior point of the sternoclavicular joint and the most posterior point of the ACJ, pointing toward ACJ, the Y axis was perpendicular to the Z axis and pointed to the cranial direction, the X axis was perpendicular to both the Y and Z axes and pointed to the anterior direction, and the origin of the coordinate system was set to the midpoint between the most anterior point of the sternoclavicular joint and the most posterior point of the ACJ on the Z axis.

## Data Availability

Not applicable.

## Abbreviations

ACJ: Acromioclavicular joint
CT: Computed tomography

## References

[1] Robinson CM (1998). Fractures of the clavicle in the adult. Epidemiol Classification J Bone Joint Surg Br.

[2] Robinson CM, Court-Brown CM, McQueen MM, Wakefield AE (2004). Estimating the risk of nonunion following nonoperative treatment of a clavicular fracture. J Bone Joint Surg Am.

[3] Frima H, van Heijl M, Michelitsch C, van der Meijden O, Beeres FJP, Houwert RM, Sommer C (2020). Clavicle fractures in adults; current concepts. Eur J Trauma Emerg Surg.

[4] Fox HM, Ramsey DC, Thompson AR, Hoekstra CJ, Mirarchi AJ, Nazir OF (2020). Neer type-II distal clavicle fractures: a cost-effectiveness analysis of fixation techniques. J Bone Joint Surg Am.

[5] Klein SM, Badman BL, Keating CJ, Devinney DS, Frankle MA, Mighell MA (2010). Results of surgical treatment for unstable distal clavicular fractures. J Shoulder Elb Surg.

[6] van der Meijden OA, Gaskill TR, Millett PJ (2012). Treatment of clavicle fractures: current concepts review. J Shoulder Elb Surg.

[7] Fleming MA, Dachs R, Maqungo S, du Plessis JP, Vrettos BC, Roche SJ (2015). Angular stable fixation of displaced distal-third clavicle fractures with superior precontoured locking plates. J Shoulder Elb Surg.

[8] Abe S, Shimada T, Murase T, Kuriyama K. Comparison of the Orientation Angles of Volar Locking Plate Distal Ulnar Locking Screw for Distal Radius Fractures. J Hand Surg [Am]. 2021, ahead of print. 10.1016/j.jhsa.2021.05.011. 10.1016/j.jhsa.2021.05.011 34158207

[9] Oka K, Murase T, Moritomo H, Goto A, Sugamoto K, Yoshikawa H (2009). Accuracy analysis of three-dimensional bone surface models of the forearm constructed from multidetector computed tomography data. Int J Med Robot.

[10] Wu G, van der Helm FCT, Veeger HEJ, Makhsous M, Van Roy P, Anglin C, Nagels J, Karduna AR, McQuade K, Wang X (2005). ISB recommendation on definitions of joint coordinate systems of various joints for the reporting of human joint motion—part II: shoulder, elbow, wrist and hand. J Biomech.

[11] Boehm TD, Kirschner S, Fischer A, Gohlke F (2003). The relation of the coracoclavicular ligament insertion to the acromioclavicular joint: a cadaver study of relevance to lateral clavicle resection. Acta Orthop Scand.

[12] Takase K (2010). The coracoclavicular ligaments: an anatomic study. Surg Radiol Anat.

[13] Neer CS (1968). Fractures of the distal third of the clavicle. Clin Orthop Relat Res.

[14] Craig E, Rockwood CA, Matson FA (1990). Fractures of the clavicle. The Shoulder.

[15] Erdle B, Izadpanah K, Jaeger M, Jensen P, Konstantinidis L, Zwingmann J, Sudkamp NP, Maier D (2017). Comparative analysis of locking plate versus hook plate osteosynthesis of Neer type IIB lateral clavicle fractures. Arch Orthop Trauma Surg.

[16] Asadollahi S, Bucknill A (2019). Hook plate fixation for acute unstable distal clavicle fracture: a systematic review and Meta-analysis. J Orthop Trauma.

[17] Yagnik GP, Brady PC, Zimmerman JP, Jordan CJ, Porter DA (2019). A biomechanical comparison of new techniques for distal clavicular fracture repair versus locked plating. J Shoulder Elb Surg.

[18] Rieser GR, Edwards K, Gould GC, Markert RJ, Goswami T, Rubino LJ (2013). Distal-third clavicle fracture fixation: a biomechanical evaluation of fixation. J Shoulder Elb Surg.

[19] Kuner E, Beeres FJP, Babst R, Schoeniger R (2019). Which lateral clavicle fractures can be treated by an arthroscopic-assisted endobutton procedure? An analysis of risk factors. Arch Orthop Trauma Surg.

[20] Fukuda K, Craig EV, An KN, Cofield RH, Chao EY (1986). Biomechanical study of the ligamentous system of the acromioclavicular joint. J Bone Joint Surg Am.

[21] Gautier E, Sommer C (2003). Guidelines for the clinical application of the LCP. Injury.

[22] Andersen JR, Willis MP, Nelson R, Mighell MA (2011). Precontoured superior locked plating of distal clavicle fractures: a new strategy. Clin Orthop Relat Res.

[23] Martetschlager F, Kraus TM, Schiele CS, Sandmann G, Siebenlist S, Braun KF, Stockle U, Freude T, Neumaier M (2013). Treatment for unstable distal clavicle fractures (Neer 2) with locking T-plate and additional PDS cerclage. Knee Surg Sports Traumatol Arthrosc.

[24] Seyhan M, Kocaoglu B, Kiyak G, Gereli A, Turkmen M (2015). Anatomic locking plate and coracoclavicular stabilization with suture endo-button technique is superior in the treatment of Neer type II distal clavicle fractures. Eur J Orthop Surg Traumatol.

[25] Yoo JH, Chang JD, Seo YJ, Shin JH (2009). Stable fixation of distal clavicle fracture with comminuted superior cortex using oblique T-plate and cerclage wiring. Injury.

[26] Wähnert D, Windolf M, Brianza S, Rothstock S, Radtke R, Brighenti V, Schwieger K (2011). A comparison of parallel and diverging screw angles in the stability of locked plate constructs. J Bone Joint Surg (Br).

[27] Robert KQ, Chandler R, Baratta RV, Thomas KA, Harris MB (2003). The effect of divergent screw placement on the initial strength of plate-to-bone fixation. J Trauma.

