# Supplementary material for: Comparing the locking screw direction of three locking plates for lateral clavicle fractures: a simulation study
Source: BMC Musculoskelet Disord. 2021 Sep 21;22:812. doi: 10.1186/s12891-021-04697-5 (PMC8456609; doi:10.1186/s12891-021-04697-5)
Supplement: Supplementary file 1 — Additional file 1: Supplementary material 1. The coordinate system of the plate. Three points on the plates were assessed: the medial and lateral end points of the plate with its midpoint of thickness, the midpoint of the length with dorsal rim, and midpoint of the thickness of the plate. The basal plane that passes through these three points was created. The Z axis passed through the medial and lateral points and pointed toward the lateral side of the plate on the basal plane. The X axis pointed toward the volar side of the plate on the basal plane perpendicular to the Z axis. The Y axis was perpendicular to both the X and Y axes and pointed toward the cranial side. The coordinate system of the clavicle. The Z axis connected the most anterior point of the sternoclavicular joint and the most posterior point of the ACJ, pointing toward ACJ, the Y axis was perpendicular to the Z axis and pointed to the cranial direction, the X axis was perpendicular to both the Y and Z axes and pointed to the anterior direction, and the origin of the coordinate system was set to the midpoint between the most anterior point of the sternoclavicular joint and the most posterior point of the ACJ on the Z axis. [file 12891_2021_4697_MOESM1_ESM.docx]

**Supplementary material 1**

**The coordinate system of the plate**

Three points on the plates were assessed: the medial and lateral end points of the plate with its midpoint of thickness, the midpoint of the length with dorsal rim, and midpoint of the thickness of the plate. The basal plane that passes through these three points was created. The Z axis passed through the medial and lateral points and pointed toward the lateral side of the plate on the basal plane. The X axis pointed toward the volar side of the plate on the basal plane perpendicular to the Z axis. The Y axis was perpendicular to both the X and Y axes and pointed toward the cranial side.

**The coordinate system of the clavicle**

The Z axis connected the most anterior point of the sternoclavicular joint and the most posterior point of the ACJ, pointing toward ACJ, the Y axis was perpendicular to the Z axis and pointed to the cranial direction, the X axis was perpendicular to both the Y and Z axes and pointed to the anterior direction, and the origin of the coordinate system was set to the midpoint between the most anterior point of the sternoclavicular joint and the most posterior point of the ACJ on the Z axis.
